# Neuroplastin Modulates Anti-inflammatory Effects of MANF

**DOI:** 10.1016/j.isci.2020.101810

**Published:** 2020-11-17

**Authors:** Takuya Yagi, Rie Asada, Kohsuke Kanekura, Ave Eesmaa, Maria Lindahl, Mart Saarma, Fumihiko Urano

**Affiliations:** 1 Department of Medicine, Division of Endocrinology, Metabolism, and Lipid Research, Washington University School of Medicine, St. Louis, MO 63110, USA; 2 Department of Pathology and Immunology, Washington University School of Medicine, St. Louis, MO 63110, USA; 3 Department of Molecular Pathology, Tokyo Medical University, Tokyo 160-8402, Japan; 4 Institute of Biotechnology, HiLIFE, University of Helsinki, Viikinkaari 9, 00014 Helsinki, Finland

**Keywords:** Biochemistry, Molecular Biology, Cell Biology

## Abstract

Endoplasmic reticulum (ER) stress is known to induce pro-inflammatory response and ultimately leads to cell death. Mesencephalic astrocyte-derived neurotrophic factor (MANF) is an ER-localized protein whose expression and secretion is induced by ER stress and a crucial survival factor. However, the underlying mechanism of how MANF exerts its cytoprotective activity remains unclear due to the lack of knowledge of its receptor. Here we show that Neuroplastin (NPTN) is such a receptor for MANF. Biochemical analysis shows the physiological interaction between MANF and NPTN on the cell surface. Binding of MANF to NPTN mitigates the inflammatory response and apoptosis via suppression of NF-kβ signaling. Our results demonstrate that NPTN is a cell surface receptor for MANF, which modulates inflammatory responses and cell death, and that the MANF-NPTN survival signaling described here provides potential therapeutic targets for the treatment of ER stress-related disorders, including diabetes mellitus, neurodegeneration, retinal degeneration, and Wolfram syndrome.

## Highlights

• Neuroplastin (NPTN) is a plasma membrane receptor for MANF
• NPTN regulates MANF-mediated suppression of inflammation
• NPTN regulates cell survival mediated by MANF under ER stress
• MANF-NPTN survival pathway provides potential therapeutic targets for ER stress-related disorders

## Graphical Abstract

**Figure:**
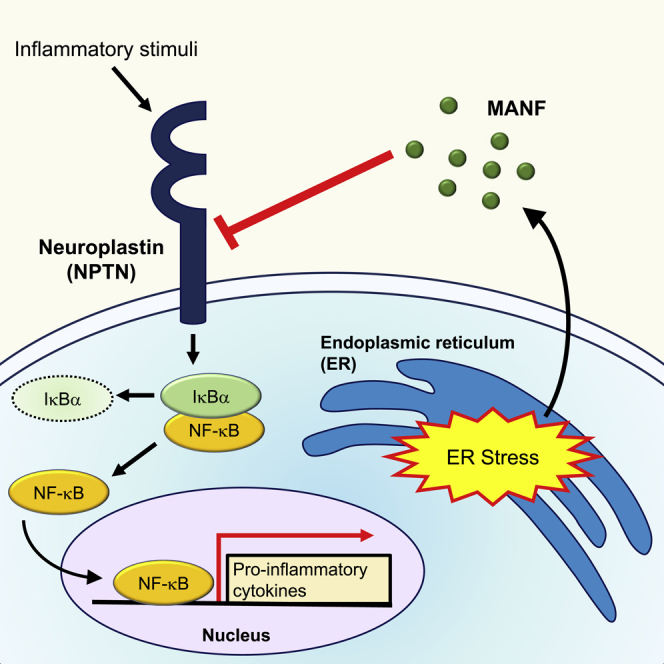

## Introduction

The endoplasmic reticulum (ER) participates in many important cellular processes. This includes the native folding, post-translational modification, and trafficking of transmembrane and secretory proteins. Genetic or acquired dysfunction of the ER leads to a variety of diseases. Imbalance between the demand for secretory and membrane proteins and the protein folding capacity of ER results in a buildup of unfolded/misfolded proteins in the ER. The status in which unfolded/misfolded proteins are accumulated in the ER lumen is referred to as ER stress. Once ER stress occurs, it is mitigated by a signaling mechanism known as the unfolded protein response (UPR) (Ron and Walter, 2007; Schroder and Kaufman, 2005). The UPR maintains ER homeostasis through three distinct physiological responses: (1) translational attenuation to decrease the demands made on the organelle (Harding et al., 2002), (2) the transcriptional induction of genes encoding ER-resident chaperones to facilitate protein folding (Li et al., 2000; Yoshida et al., 1998), and (3) ER-associated degradation (ERAD) to degrade unfolded or misfolded proteins in the ER lumen (Ng et al., 2000; Travers et al., 2000). Excessive and unresolved ER stress induces pro-inflammatory response and eventually leads to apoptotic cell death, which contributes to the pathogenesis of a variety of diseases including neurodegenerative disorders and diabetes mellitus (Nakagawa et al., 2000; Urano et al., 2000).

MANF is an ER stress responsive protein whose expression and secretion are enhanced by ER stress (Apostolou et al., 2008; Mizobuchi et al., 2007). MANF together with cerebral dopamine neurotrophic factor (CDNF) was originally identified as highly evolutionarily conserved neurotrophic factors (NTFs) forming a novel family of NTFs (Lindahl et al., 2017; Lindholm and Saarma, 2010; Lindholm et al., 2007; Petrova et al., 2003). Traditionally, it was considered that there were three families of NTFs: (1) neurotrophin family, (2) glial cell line-derived neurotrophic factor (GDNF) family of ligands (GFLs), and (3) neurotrophic cytokines (neurokines). By binding to and activating their receptors on the cell surface, NTFs transmit cytoprotective and survival signals via a phosphorylation cascade (Airaksinen et al., 1999; Chao, 2003). MANF and CDNF are structurally distinct from all other families of NTFs and signal through unknown receptors or mechanisms (Lindahl et al., 2017).

Previous studies demonstrated that MANF is a protective factor for dopamine neurons in animal models of Parkinson disease, cardiac myocytes in myocardial infarction, cortical neurons in ischemic stroke, and retinal cells in models of photodamage (Airavaara et al., 2009; Glembotski et al., 2012; Lu et al., 2018; Neves et al., 2016; Voutilainen et al., 2009). MANF is indispensable for the survival of pancreatic β-cells (Lindahl et al., 2014). Negative regulation of nuclear factor (NF)-κB has been shown as cytoprotective effect of MANF (Chen et al., 2015b; Hakonen et al., 2018). However, the precise signaling pathways regulated by MANF remain unclear.

As NTFs are known to bind to cell surface receptors to activate downstream signaling, a specific receptor for MANF is expected to exist. In this study, we show that neuroplastin (NPTN) is such a cell surface receptor for MANF. NPTN mediates the expression and secretion of inflammatory cytokines through activation of the NF-κB pathway, and MANF antagonizes the inflammatory effect of NPTN by direct physical interaction, resulting in the suppression of ER stress-mediated inflammation and cell death. Our study reveals a mechanism of MANF-mediated cell survival and anti-inflammation.

## Results

### An Unbiased Cell Surface Binding Assay to Identify MANF Receptor Candidates

**Figure 1:**
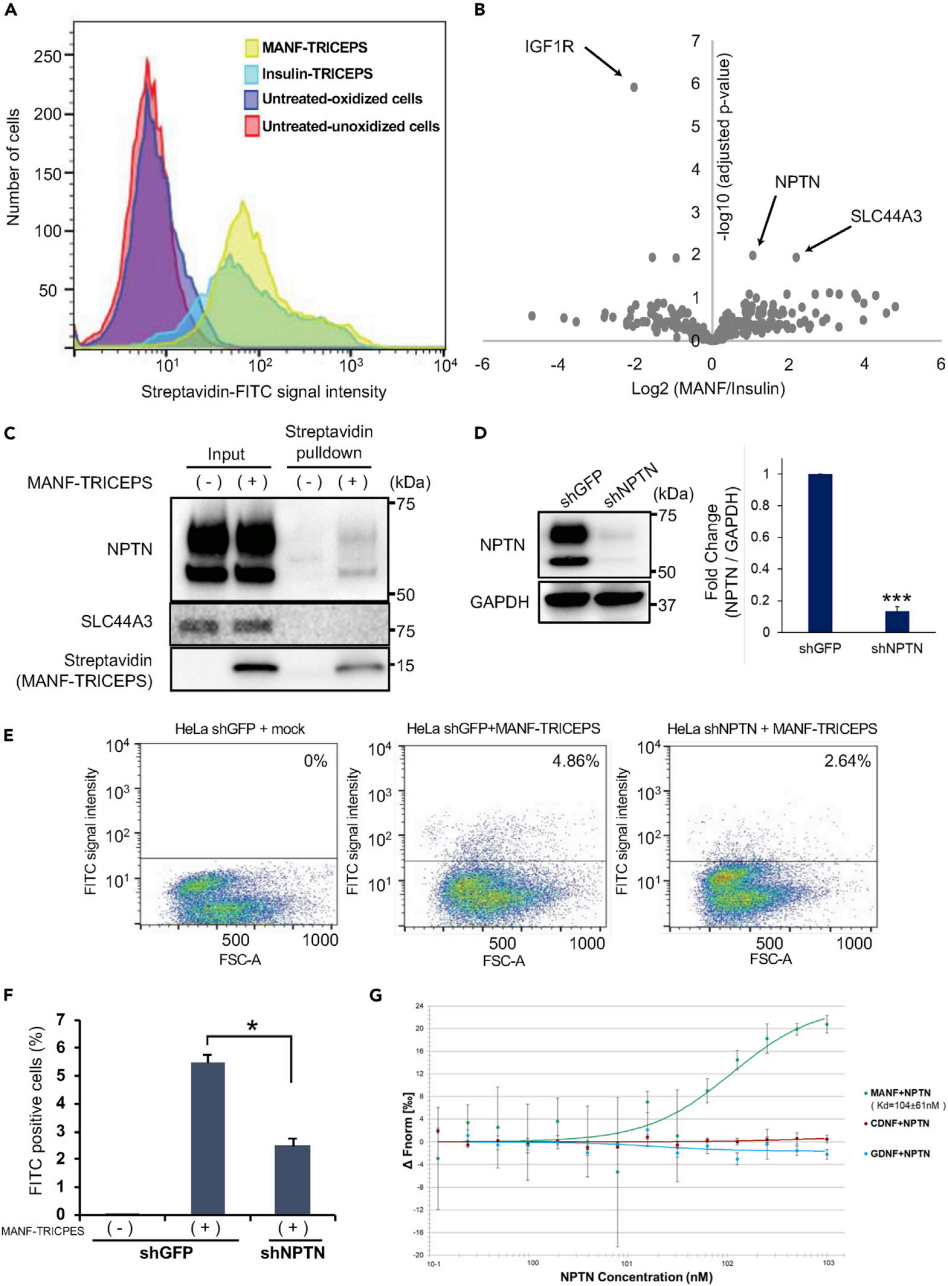
Identification of MANF Receptor by a Ligand Receptor Capture (LRC) Technology (A) Fluorescence-activated cell sorting (FACS) analysis examining the binding and cross-linking of MANF peptide-TRICEPS and Insulin peptide-TRICEPS to the oxidized glycans on the cell surface of INS-1 832/13 cells. (B) CaptiRec volcano plot to compare enriched proteins analyzed by liquid chromatography-tandem mass spectrometry (LC-MS/MS) in the Insulin and MANF peptide samples. Data are shown on the protein level. (C) Immunoblot analysis monitoring NPTN and MANF-TRICEPS in HeLa cells treated with mock (−) or MANF-TRICEPS (+). Lysates from HeLa cells were pulled down with streptavidin beads. (D) The evaluation of NPTN knockdown with lentivirus expressing shGFP or shNPTN in HeLa cells. Left panel is a representative immunoblot image of NPTN and GAPDH. GAPDH was used as a loading control. Right graph is quantitative analysis of NPTN from three independent experiments (n = 3; values are mean ± SD, ∗∗∗p < 0.001, unpaired two-tailed Student's t test). (E and F) HeLa cells stably expressing shGFP or shNPTN were mixed with MANF-TRICEPS and were examined by FACS to investigate the binding. Representative results of FACS analyses (E). The graph shows the average percentage of FITC-positive cells of three independent experiments (F) (n = 3; values are mean ± SD, ∗p < 0.05, one-way ANOVA followed by Dunnett's test). (G) For the microscale thermophoresis, recombinant MANF, CDNF, and GDNF polypeptides were fluorescently labeled with Alexa 647, and the concentration of labeled molecules was kept constant at 10 nM in all runs. Recombinant NPTN polypeptide was titrated over a range of concentrations from 0.122 to 1,000 nM. Shown are the mean ΔFnorm-values ± SD resulting from n = 3 independent repeats. The means are fitted using Nanotemper MO.Affinity analysis v2.1.2 assuming a 1:1 binding stoichiometry and the resulting K_d_ value is given together with an error estimation from the fit.

To identify cell surface molecules that bind to the extracellular MANF in mammalian cells, we adapted a ligand-receptor capture system TRICEPS (Frei et al., 2012). This system utilizes a specifically designed chemoproteomic reagent TRICEPS, which has three heads: one is covalently coupled to a ligand of interest, one is used for cross-linking to oxidized glycan of the target receptor, and one is modified with biotin for purification with streptavidin. MANF protects pancreatic β-cells from ER stress-mediated cell death *in vitro* and *in vivo* (Lindahl et al., 2014). Therefore, we hypothesized that pancreatic β-cells might have abundant MANF receptor expression. To test this idea, we used MANF and insulin polypeptides as ligands to capture receptor candidates in a rat β-cell line INS-1 832/13 cells. The receptor capturing efficiency was monitored by flow cytometric analysis of fluorescein isothiocyanate (FITC)-labeled streptavidin. As shown in Figure 1A, the robust fluorescent signal of INS-1 832/13 cells labeled by FITC-streptavidin indicates that MANF-TRICEPS, as well as Insulin-TRICEPS, bound to cell surface molecules efficiently. After purification with streptavidin beads, samples were analyzed by liquid chromatography-tandem mass spectrometry (LC-MS/MS). 882 glycopeptides were identified and quantified in our experiments. Among those, 269 glycoproteins were known to be localized to the cell membrane. As expected, Insulin-TRICEPS captured insulin-like grown factor 1 (IGF1) receptor, one of the known insulin receptors, indicating that this LRC screen worked properly (Varewijck and Janssen, 2012). MANF-TRICEPS sample captured two receptor candidates, neuroplastin (NPTN) and SLC44A3 (also known as CTL3) (Figure 1B). To confirm the binding between MANF and these candidate receptors, we conducted a pull-down assay. The assay revealed that MANF interacted with NPTN, but not with SLC44A3 (Figure 1C). NPTN knockdown by short hairpin RNA (shRNA) in HeLa cells significantly reduced the signal of the cells labeled by FITC-streptavidin (Figures 1D–1F), suggesting that MANF interacts with NPTN *in vivo*. Because CDNF has a structural similarity with MANF, we tested the ability of CDNF to bind NPTN using microscale thermophoresis. MANF, CDNF, and GDNF (negative control) polypeptides were titrated with varying concentrations of NPTN polypeptide. We found strong binding of NPTN to MANF, but not to CDNF or GDNF, indicating that NPTN is a specific binding partner with MANF. Based on these analyses, we focused on NPTN to verify if NPTN is a receptor for MANF.

### NPTN Is Upregulated under ER Stress

**Figure 2:**
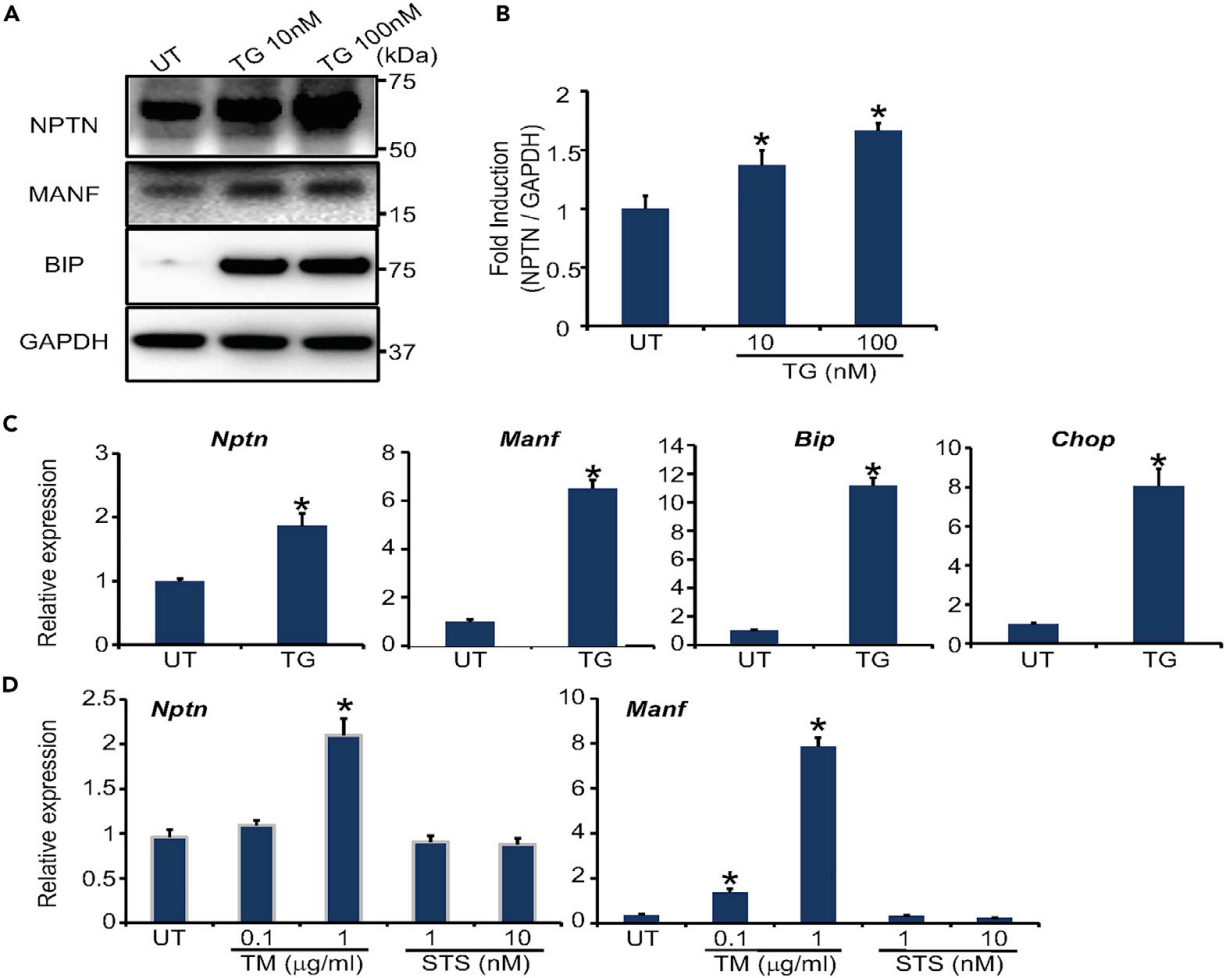
NPTN Expression Is Increased by ER Stress (A) Representative immunoblot images of NPTN, MANF, BiP, and GAPDH in C6 cells treated with thapsigargin (TG, 10 nM and 100 nM) for 16 h or untreated (UT). GAPDH was used as a loading control. (B) Quantitative analysis of NPTN expression. The number is the average of three independent experiments (n = 3; values are mean ± SD, ∗p < 0.05, one-way ANOVA followed by Dunnett's test). (C) qPCR analysis of *Nptn*, *Manf*, *Bip,* and *Chop* in C6 cells treated with 10 nM TG for 16 h or untreated (UT) (n = 3; values are mean ± SD., ∗p < 0.05, unpaired two-tailed Student's t test). (D) qPCR analysis of *Nptn* and *Manf* in C6 cells treated with tunicamycin (TM, 0.1 μg/mL and 1 μg/mL), staurosporin (STS, 0.01 nM and 0.1 nM) for 16 h or untreated (UT) (n = 3; values are mean ± SD, ∗p < 0.05, one-way ANOVA followed by Dunnett's test).

First, we sought to determine whether NPTN expression could be increased by ER stress because MANF expression is known to be increased under ER stress conditions (Apostolou et al., 2008; Mizobuchi et al., 2007). Rat glial C6 cells were treated with thapsigargin, a chemical ER stressor. NPTN mRNA and protein and MANF mRNA levels were increased just like as other known ER stress marker genes (*BiP* and *CHOP*) (Figures 2A and 2B). Another ER stressor, tunicamycin, also increased mRNA levels of *NPTN* and *MANF*. On the other hand, this increase was not observed by treatment with staurosporin, an agent that induces apoptosis independently of ER stress, suggesting that *NPTN* is specifically upregulated by ER stress (Figure 2C and 2D).

### NPTN Induces Inflammation via NF-κB Activation

**Figure 3:**
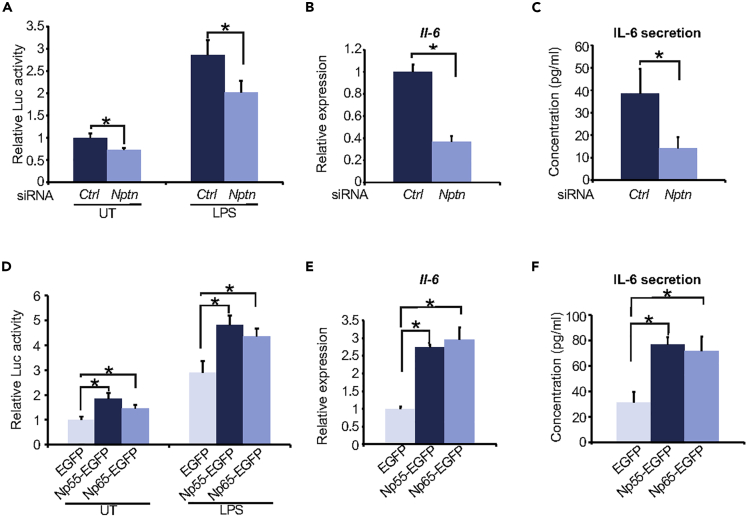
NPTN Activates Inflammatory Response through the NF-κB Pathway (A–C) (A) Luciferase assay in C6 cells cotransfected with NF-κB luciferase, pRL-TK, and either a scrambled siRNA (si*Ctrl*) or siRNA targeting rat *Nptn* (si*Nptn*). At 48 h after transfection, the cells were treated with LPS 100 ng/mL for 8 h and then the luciferase activity was measured (n = 6; values are mean ± SD, ∗p < 0.05, one-way ANOVA followed by Dunnett's test). (B and C) C6 cells were transiently transfected with si*Ctrl* or si*Nptn*. At 48 h after transfection, mRNA level (B) or secretion level (C) of IL-6 was measured by qPCR or ELISA (n = 3; values are mean ± SD, ∗p < 0.05, unpaired two-tailed Student's t test). (D–F) (D) C6 cells were cotransfected with NF-κB luciferase, pRL-TK, and a vector expressing EGFP, Np55-EGFP, or Np65-EGFP. At 48 h after transfection, the cells were treated with LPS 100 ng/mL for 8 h and then the luciferase activity was measured (n = 6; values are mean ± SD, ∗p < 0.05, one-way ANOVA followed by Dunnett's test). (E and F) C6 cells were transiently transfected with a vector expressing EGFP, Np55-EGFP, or Np65-EGFP. At 48 h after transfection, the gene expression level (E) or secretion level (F) of IL-6 was measured by qPCR or ELISA (n = 3; values are mean ± SD, ∗p < 0.05, one-way ANOVA followed by Dunnett's test).

Previous studies demonstrate that MANF negatively regulates NF-κB, thus reducing inflammation (Chen et al., 2015b; Hakonen et al., 2018). Indeed, when we performed knockdown of *Manf* in C6 cells using small interfering RNA (siRNA) (Figures S1A and S1B), *Manf* knockdown cells showed increased NF-κB activation (Figure S1C). Consistent with this finding, mRNA and secretion levels of proinflammatory cytokine, interleukin (IL)-6, and CXCL-1 (ortholog of human IL-8) were increased in *Manf* knockdown cells (Figures S1D–S1F). Therefore, we tested if NPTN might be involved in inflammation and NF-κB activation. Treatment of C6 cells with lipopolysaccharide (LPS) robustly increased NF-κB-dependent luciferase activity in control, whereas *Nptn* knockdown suppressed the luciferase activity both in untreated and LPS-treated cells (Figures 3A, S2A, and S2B). In addition, *Nptn* knockdown significantly reduced mRNA and secretion levels of IL-6 (Figures 3B and 3C). We next conducted overexpression of *Nptn* using Np55-EGFP (a shorter isoform of NPTN) or Np65-EGFP (a longer isoform of NPTN) vector (Figure S1C). Contrary to knockdown experiments, transient overexpression of Np55 and Np65 significantly induced NF-κB-dependent luciferase activity, as well as mRNA and secretion levels of IL-6 (Figures 3D–3F). Collectively, these results indicate that NPTN activates inflammatory responses through NF-κB signaling.

### MANF Suppresses NF-κB Activation through NPTN

**Figure 4:**
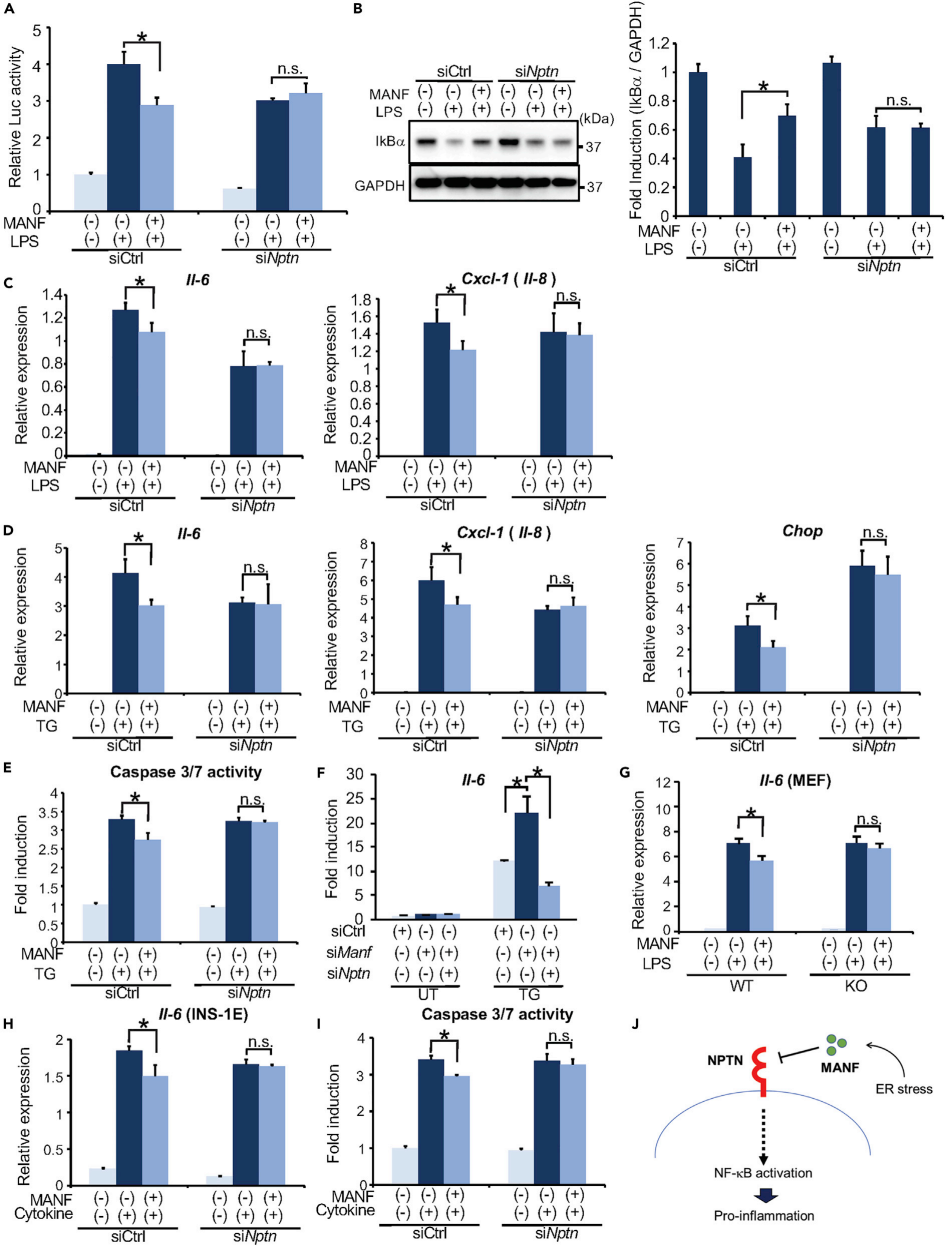
MANF Inhibits NF-κB Pathway via NPTN (A) Luciferase assay with C6 cells cotransfected with NF-κB luciferase, pRL-TK, and either si*Ctrl* or si*Nptn.* At 48 h after transfection, the cells were pre-treated with or without MANF (3 μg/mL for 1 h) before LPS treatment (100 ng/mL for 8 h) and then the luciferase activity was measured (n = 6; values are mean ± SD, ∗p < 0.05). (B) Representative immunoblot images of IκBα and GAPDH with C6 cells transfected with si*Ctrl* or si*Nptn.* At 48 h after transfection, the cells were pre-treated with MANF (3 μg/mL for 1 h) before LPS treatment (100 ng/mL for 30 min) and then lysed. The right graph indicates quantification of IκBα protein levels. The number is the average of three independent experiments. GAPDH was used as a loading control. (n = 3; values are mean ± SD, ∗p < 0.05). (C) qPCR analysis of *Il-6* and *Cxcl-1* (*Il-8*) using C6 cells transfected with si*Ctrl* or si*Nptn*. At 48 h after transfection, the cells were pre-treated with or without MANF (3 μg/mL for 1 h) before LPS treatment (100 ng/mL for 8 h) (n = 3; values are mean ± SD, ∗p < 0.05). (D) qPCR analysis of *Il-6*, *Cxcl-1* (*Il-8*), and *Chop* using C6 cells transfected with si*Ctrl* or si*Nptn*. After 48 h of transfection, the cells were pre-treated with or without MANF (3 μg/mL for 1 h) before TG treatment (100 nM for 8 h) (n = 3; values are mean ± SD, ∗p < 0.05). (E) C6 cells were treated with TG (100 nM, for 8 h) 48 h after transfection with si*Ctrl* or si*Nptn*. Caspase 3/7 activity was monitored by a Promega Caspase-Glo 3/7 kit (n = 6; values are mean ± SD, ∗p < 0.05). (F) qPCR analysis of *Il-6* using C6 cells transfected with si*Ctrl*, si*Manf,* or si*Manf* and si*Nptn*. At 48 h after transfection, the cells were treated with or without TG (100 nM, for 8 h) (n = 3; values are mean ± SD, ∗p < 0.05). (G) qPCR analysis of *Il-6* in primary cultured MEF from wild-type (WT) or *Nptn* knockout (KO) mice. The cells were pre-treated with or without MANF (3 μg/mL for 1 h) before LPS treatment (100 ng/mL for 8 h) (n = 3; values are mean ± SD, ∗p < 0.05). (H) qPCR analysis of *Il-6* with INS-1E cells. The cells were treated with cytokine cocktail (IL-1β and IFN-γ 50 ng/mL for 8 h) 48 h after transfection with si*Ctrl* or si*Nptn* (n = 3; values are mean ± SD, ∗p < 0.05). (I) INS-1E cells were treated with cytokine cocktail (IL-1β and IFN-γ 50 ng/mL for 8 h) 48 h after transfection with si*Ctrl* or si*Nptn*, then caspase 3/7 activity was monitored by a Promega Caspase-Glo 3/7 kit (n = 6; values are mean ± SD, ∗p < 0.05). (J) Schematic of the relationship between NPTN and MANF. Statistical analysis of data was performed by one-way ANOVA followed by Dunnett's test.

Given the direct binding between MANF and NPTN, we tested if MANF might suppress the NF-κB activity through NPTN. First, we investigated the dynamics of this interaction in the presence of ER stress or inflammatory stimuli. After co-transfecting Np65-EGFP and MANF expression vectors in HeLa cells, we treated these cells with or without thapsigargin or LPS. The cell lysates were immunoprecipitated with anti-GFP antibody and subsequently immunoblotted. The amount of MANF interacting with NPTN was not changed by the treatment with either thapsigargin or LPS, indicating that ER stress and inflammatory stimuli do not affect the interaction between NPTN and MANF (Figure S3). Consistent with previous reports and our observations (Figure S1), co-treatment with recombinant MANF polypeptide and LPS reduced NF-κB-dependent luciferase activity compared with LPS alone. This reduction was blunted in *Nptn* knockdown cells (Figure 4A). Under basal conditions, NF-κB complexes with IκB, which is a physiological inhibitor for NF-κB. Under inflammatory conditions, IκB is degraded by the ubiquitin-proteasome system. This is followed by the release of active NF-kB, which promotes transcription of inflammatory cytokines (Afonina et al., 2017). LPS stimulation decreased the protein levels of IκB, which was restored by MANF (Figure 4B). Knockdown of *Nptn* negated the effects of MANF on IκB protein levels. Furthermore, MANF treatment significantly suppressed the expressions of the well-established NF-κB target genes, *IL-6* and *Cxcl-1* (*IL-8*). Knockdown of *Nptn* attenuated this effect (Figures 4C and 4D). As reported previously, ER stress induces inflammation leading to cell death (Hotamisligil, 2010). Thapsigargin treatment increased not only mRNA expression levels of *Il-6* and *Cxcl-1* (*Il-8*) but also those of *Chop*, which is a major molecule in ER stress-induced cell death (Figure 4D). Additionally, caspase 3/7 activity was increased by thapsigargin treatment (Figure 4E). As expected, recombinant MANF polypeptide suppressed expression levels of ER stress marker genes, NF-κB activation, and cell death mediated by thapsigargin. Consistent with those results, MANF-mediated suppression of ER stress, NF-kB activation, and cell death was not observed in *Nptn* knockdown cells. Furthermore, Manf knockdown in C6 cells increased the expression of NF-kB target gene, Il-6, induced by an ER stressor, thapsigargin. In contrast, the simultaneous knockdown of Manf and Nptn negates this increase. These results indicate that ER stress-induced MANF suppresses NF-kB activation via NPTN (Figure 4F). MANF-mediated suppression of Il-6 was also not observed in *Nptn* knockout mouse embryonic fibroblasts (Figure 4G). Cytokines are potent inducers of ER stress and are known to play a critical role in the autoimmune destruction of islets in type 1 diabetes mellitus (T1D) (Kutlu et al., 2003; Lopes et al., 2014). Moreover, *Manf* knockout mice spontaneously develop diabetes mellitus due to β-cell death within pancreatic islets (Lindahl et al., 2014). These previous reports and our findings motivated us to hypothesize that MANF can protect islets from β-cell death via binding to NPTN in T1D. To verify our hypothesis, we employed *in vitro* model of T1D. INS-1E cells were treated with cytokine cocktail (IL-1β and IFN-γ 50 ng/mL), and we then monitored caspase 3/7 activity and *Il-6* mRNA levels. Treatment with recombinant MANF polypeptide modestly attenuated cytokine-mediated cell death with suppression of IL-6 expression. As expected, knockdown of *Nptn* abrogated this protective effect (Figures 4H, 4I, and S4). Collectively, these findings suggest that MANF alleviates inflammation, ER stress, and cell death by binding to NPTN in a variety of cell types.

## Discussion

MANF was originally identified as the new family member of NTFs. Further studies revealed that MANF is highly expressed in non-neuronal tissues and circulates in the blood (Lindahl et al., 2017). MANF signaling is an emerging therapeutic target in neurodegenerative diseases, diabetes mellitus, stroke, retinal damage, and Wolfram syndrome, a genetic disorder characterized by diabetes and retinal and neuronal degeneration. However, the plasma membrane receptor for MANF involved in cytoprotective and anti-inflammatory effects was not identified. In this study, we show that NPTN is such a receptor for MANF.

Several previous studies demonstrated that MANF inhibits inflammation (Chen et al., 2015a; Liu et al., 2019; Neves et al., 2016). Our study demonstrates that NPTN has pro-inflammatory effects that are suppressed by MANF. Recently, the pro-inflammatory secretory proteins, S100A8 and S100A9, bind to two isoforms of NPTN, Np65 and Np55, respectively (Sakaguchi et al., 2016). S100A9 is also reported to exclusively bind to CD147, which induces inflammation by recruiting TRAF2 to the cytoplasmic domain of CD147, leading to subsequent NF-κB activation (Hibino et al., 2013). CD147 has a high structural similarity with Np55, and both proteins have Ig2 and Ig3 domains. Interestingly, the cytoplasmic domain of NPTN is able to interact with TRAF2, and NPTN heterodimerizes with CD147 (Beesley et al., 2014; Sakaguchi et al., 2016). These reports and our present study suggest that the anti-inflammatory effects of MANF may be mediated by the inhibition of S100A9 and/or TRAF2.

ER stress-mediated inflammation is a promising target for preventing β-cell death in diabetes (Clark and Urano, 2016; Lerner et al., 2012; Oslowski et al., 2012; Papa, 2012). MANF-deficient mice develop diabetes due to ER stress-mediated pancreatic β-cell death (Lindahl et al., 2014). Furthermore, increased circulating MANF levels have been detected in the sera of patients with type 1 and type 2 diabetes (Galli et al., 2016; Wu et al., 2017). Our data demonstrate that MANF mitigates inflammation and cell death by suppressing the NPTN-mediated inflammatory signal in a cell model of type 1 diabetes. In addition to diabetes mellitus, cytoprotective effects of MANF have also been shown in animal models of Parkinson disease, ischemia, and retinal neurodegenerative diseases (Airavaara et al., 2009; Glembotski et al., 2012; Lu et al., 2018; Neves et al., 2016; Voutilainen et al., 2009). Furthermore, we have recently reported that MANF prevents ER stress-mediated β-cell death in cell and mouse models of Wolfram syndrome (Mahadevan et al., 2020). These previous reports and our findings indicate that screening assays, such as surface plasmon resonance (SPR), to discover small molecules that bind to NPTN and mediate survival and anti-inflammatory effects will lead to a novel therapeutic strategy for a variety of ER stress-related diseases, including diabetes, neurodegeneration, retinal degeneration, and Wolfram syndrome (Abreu and Urano, 2019).

Ca^2+^ homeostasis is known to play a pivotal role in fine-tuning insulin release from pancreatic β-cells. ER dysfunction, in particular ER stress, leads to disruptions in Ca^2+^ homeostasis that interfere with β-cell function (Clark and Urano, 2016; Marre and Piganelli, 2017). The key molecules linking ER homeostasis and Ca^2+^ signaling are not clear. Our study has revealed that ER stress responsive protein MANF physically interacts with NPTN. Recent reports demonstrated that NPTN regulates intracellular Ca^2+^ homeostasis by stabilizing plasma membrane Ca^2+^ ATPases (PMCAs) (Korthals et al., 2017; Schmidt et al., 2017). CD147 has been shown to be an essential subunit of the PMCA complex and a key regulator of Ca^2+^ clearance (Schmidt et al., 2017). In addition, MANF has been reported to revert phosphorylation of ryanodine receptor 2 (RyR2) leading the inhibition of Ca^2+^ leakage from the ER (Park et al., 2019). Although downstream of the interaction between MANF and NPTN remains unclear, both these molecules may contribute to the regulation of β-cell function, especially maintaining Ca^2+^ homeostasis.

Here, we report the discovery of NPTN as a plasma membrane receptor for MANF. Our findings indicate that NPTN is involved in the intercellular regulation of inflammation and cell survival mediated by MANF (Figure 4J). It has been shown that MANF interacts with BiP in the ER and regulates protein-folding homeostasis (Yan et al., 2019). Because MANF is also located in the lumen of the ER, it is possible that there are other binding partners for MANF exerting its survival and anti-inflammatory effects. Nevertheless, our study paves the way to develop MANF-based therapies for human disorders, including β cell death in type 1 and type 2 diabetes, neurodegenerative diseases, and Wolfram syndrome characterized by juvenile onset-diabetes, optic nerve atrophy, and neurodegeneration.

### Limitations of the Study

In this article, we show that NPTN is a plasma membrane receptor for MANF and regulates intercellular regulation of inflammation and cell survival mediated by MANF under ER stress conditions. Although steady-state interaction between MANF and NPNT is shown in Figures 1C and 1G, the dynamics of this interaction in the presence of ER stress inducers and inflammatory stimuli are still not clear and should be investigated further (Figure S3).

### Resource Availability

#### Lead Contact

Further information and requests for resources and reagents should be directed to and will be fulfilled by the Lead Contact, Fumihiko Urano (urano@wustl.edu).

#### Materials Availability

All cell lines and reagents generated in this study are available from the Lead Contact with a completed Materials Transfer Agreement.

#### Data and Code Availability

All data are included in the published article and the Supplemental Information, and any additional information will be available from the lead contact upon request.

## Methods

All methods can be found in the accompanying Transparent Methods supplemental file.

## Supplemental Information

Document S1. Transparent Methods and Figures S1–S4
