# Supplementary material for: Neuroplastin Modulates Anti-inflammatory Effects of MANF
Source: iScience. 2020 Nov 17;23(12):101810. doi: 10.1016/j.isci.2020.101810 (PMC7702011; doi:10.1016/j.isci.2020.101810)
Supplement: Document S1. Transparent Methods and Figures S1–S4 [file mmc1.pdf]

iScience, Volume *23*

Supplemental Information

Neuroplastin Modulates

Anti-inflammatory Effects of MANF

Takuya Yagi, Rie Asada, Kohsuke Kanekura, Ave Eesmaa, Maria Lindahl, Mart Saarma, and Fumihiko Urano

Figure S1, Related to Figure 3.

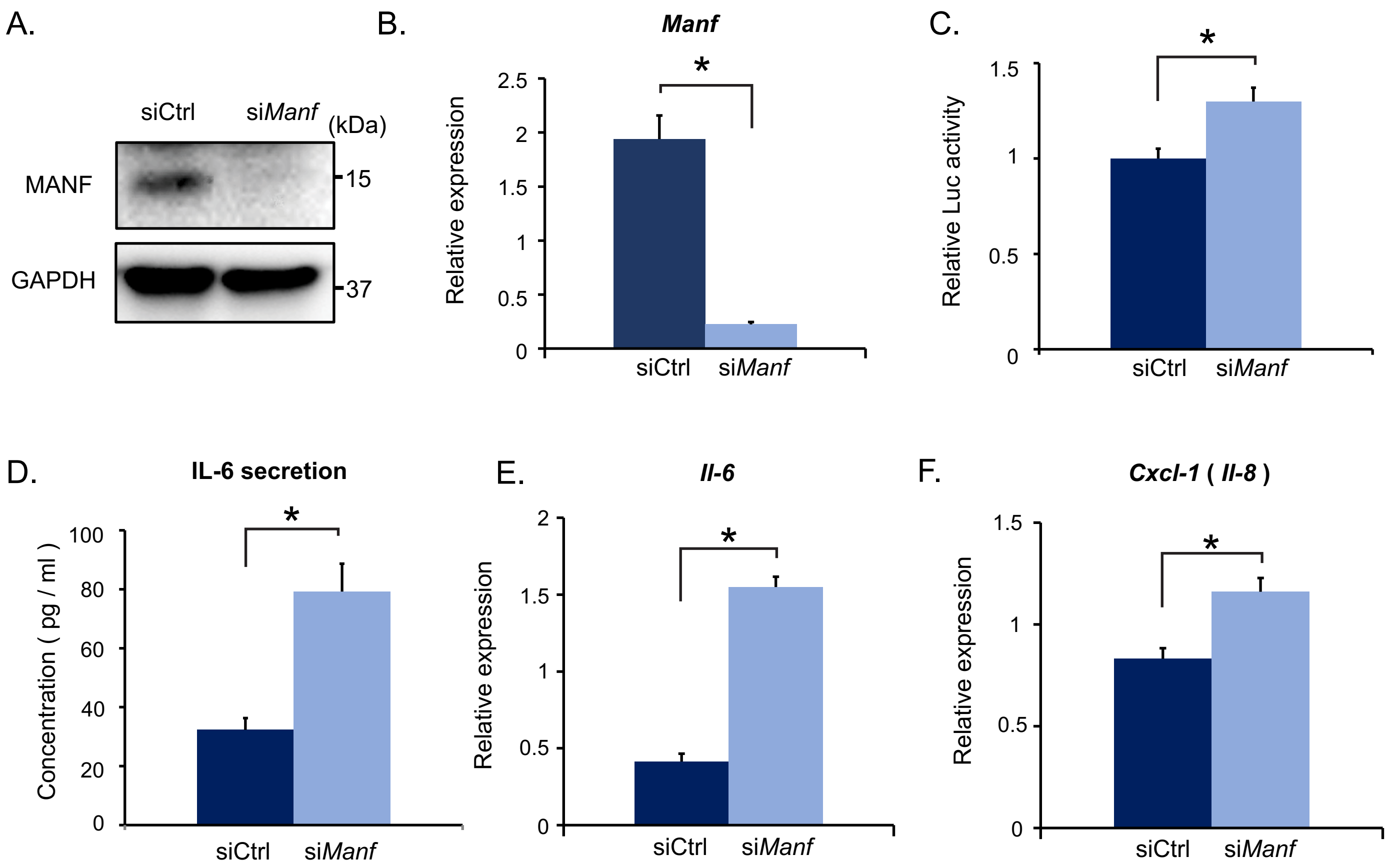

Figure S1. *Manf* knockdown induces NF-κB activation, Related to Figure 3.
(A, B) C6 cells were transiently transfected with scrambled siRNA (si*Ctrl*) or siRNA targeting rat *Manf* (si*Manf*). Protein and mRNA levels of MANF were measured by immunoblot (A) and qPCR (B). GAPDH was used as a loading control (n=3; values are mean±S.D., *P < 0.05). (C) Luciferase assay with C6 cells co-transfected with NF-κB luciferase, pRL-TK and either si*Ctrl* or si*Manf*. At 48 h after transfection, the cells were treated with LPS 100 ng/ml for 8 hours, then the luciferase activity was measured (n=6; values are mean±S.D., *P < 0.05). (D) qPCR analysis of *Il-6* and *Cxcl-1* (*Il-8*) using C6 cells transfected with si*Ctrl* or si*Manf* (n=3; values are mean±S.D., *P < 0.05). (E) ELISA of IL-6 secreted from C6 cells transfected with si*Ctrl* or si*Manf* (n=3; values are mean±S.D., *P < 0.05). Statistical analysis of data was performed by unpaired two-tailed Student's *t* test.

Figure S2, Related to Figure 3.

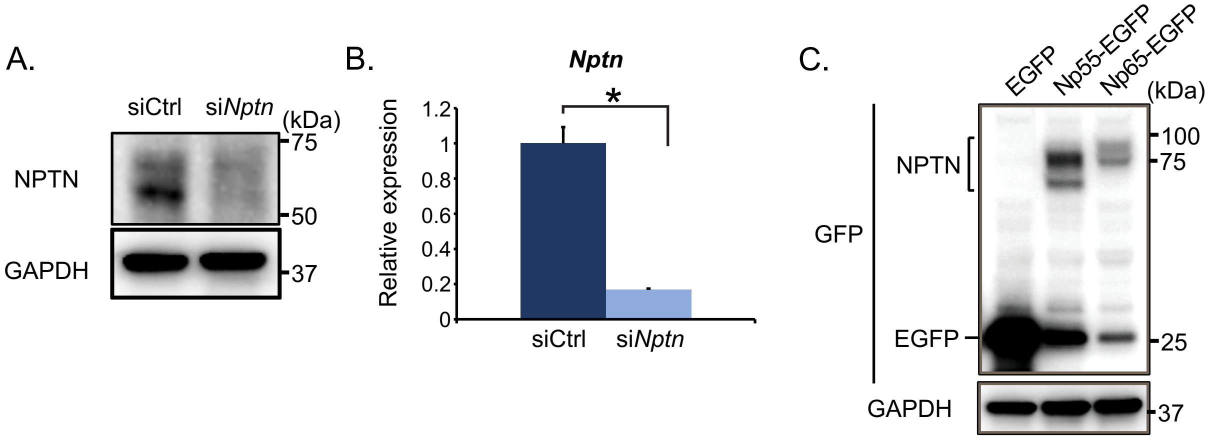

Figure S2. *Nptn* knockdown and overexpression in C6 cells, Related to Figure 3.
(A, B) C6 cells were transiently transfected with scrambled siRNA (si*Ctrl*) or siRNA targeting rat *Nptn* (si*Nptn*). Protein and mRNA levels of *Nptn* were measured by immunoblot (A) and qPCR (B). GAPDH was used as a loading control (n=3; values are mean ± S.D., *P < 0.05). (C) C6 cells were transiently transfected with a vector expressing EGFP, Np55-EGFP or Np65-EGFP. Immunoblot analysis reveals the expression of EGFP and EGFP-fused Np55 / Np65 proteins detected by GFP antibody. GAPDH was used for loading control. Statistical analysis of data was performed by unpaired two-tailed Student's *t* test.

Figure S3, Related to Figure 4.

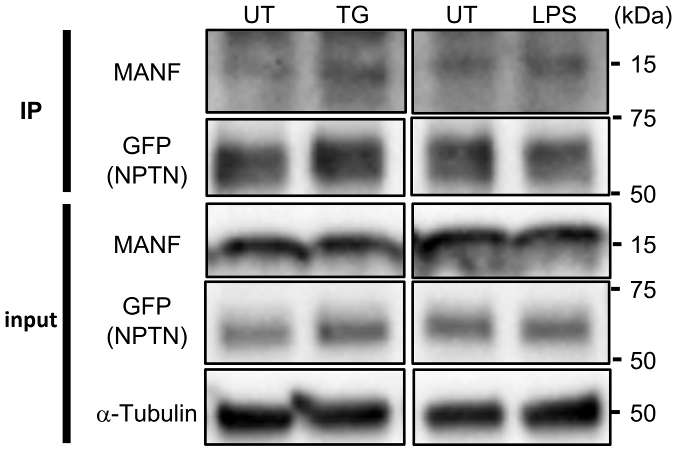

Figure S3. Treatment with TG or LPS dose not affect the interaction between MANF and NPTN, Related to Figure 4.

HeLa cells were transiently co-transfected with vectors expressing Np65-EGFP and MANF. NPTN was immunoprecipitated by anti-GFP antibody after treatment with TG (100nM) or LPS (100 ng/ml) for 8 hours, then immunoblot analysis was performed. The blot is a representative image from 3 independent experiments. Note that neither treatment affects the interaction of MANF with NPTN. α-Tubulin was used as a loading control.

Figure S4, Related to Figure 4.

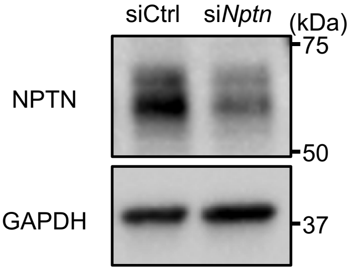

Figure S4. ***Nptn*** **knockdown in INS-1E cells, Related to Figure 4.**
INS-1E cells were transiently transfected with scrambled siRNA (si*Ctrl*) or siRNA targeting rat *Nptn* (si*Nptn*). Protein were measured by immunoblot. GAPDH was used as a loading control.

Transparent Methods

Ligand–receptor capture

TriCEPS^{TM}-based ligand-receptor capture (LRC) assay was performed utilizing CaptiRec (Dualsystems Biotech AG) to identify cell surface binding proteins for MANF of INS-1 832/13 cells according to manufacture's protocol. Briefly, TriCEPS^{TM} was coupled with 300 μg of MANF peptide (Peprotech) or human insulin as a control respectively by incubating for 2 hours at 20 ℃. Next, to gently oxidize the cell surface proteins of INS-1 832/13 cells, 1.5 mM $NaIO_4$ was added to 1 x 10^9 cell suspension. After incubation at 4 ℃ in the dark for 15 min, the cells were washed twice and then resuspended in PBS (pH 6.5). For the receptor capture reaction, the oxidized cells were treated with 100 μg of TriCEPS^{TM}-MANF / Insulin for 90 min at 4 ℃ under constant agitation. After collecting an aliquat from each sample for FACS analysis to confirm the receptor capturing efficiency, the cells were collected and the cell pellets were sent to Dualsystems Biotech AG for LC-MS/MS analysis.

The samples were analyzed on a Thermo LTQ Orbitrap XL spectrometer fitted with an electrospray ion source. The samples were measured in data dependent acquisition mode in a 40 min gradient using a 10cm C18. The nine individual samples in TriCEPS^{TM}-MANF / Insulin dataset were analyzed with a statistical ANOVA model. This model assumes that the measurement error follows Gaussian distribution and views individual features as replicates of a protein's abundance and explicitly accounts for this redundancy. It tests each protein for differential abundance in all pairwise comparisons of MANF and Insulin samples and reports the p-values. Next, p- values are adjusted for multiple comparisons to control the experiment-wide false discovery rate (FDR). The adjusted p-value obtained for every protein is plotted against the magnitude of the fold enrichment between the two experimental conditions. The area in the

volcano plot that is limited by an enrichment factor of 2 fold or greater and an FDR-adjusted p-value less than or equal to 0.05 is defined as the receptor candidate space.

Flow cytometric analyses

After incubating cells with TriCEPS–ligand complexes, cells were washed twice and stained with streptavidin-FITC (BD Biosciences) in FACS buffer (1% FBS in PBS) for 20 min. Labeled cells were washed once and analyzed with LSR II (BD Biosciences). The results were analysed by Flowjo X (10.0.7r2) software.

Coimmunoprecipitation with TriCEPS–ligand complexes

After incubating cells with TriCEPS–ligand complexes, cells were lysed in cell lysis buffer (150 mM NaCl, 0.5 % NP40, 50 mM HEPES pH 7.4, 1 mM EDTA and protease inhibitor cocktail (Roche)) followed by centrifugation at 15,000 rpm to remove cellular debris. The supernatants were mixed with Streptavidin (Cell Signaling) and incubated at 4°C for 4 hours. The beads were washed four times with the cell lysis buffer and analyzed by SDS-PAGE and immunoblot analysis.

Microscale thermophoresis

Recombinant MANF (P-101-100, Icosagen), CDNF (Biovian) and GDNF (P-103-100, Icosagen) were fluorescently labeled with Alexa647 using the Monolith Protein Labeling Kit RED-NHS following manufacturer's instructions (MO-L001, Nanotemper Technologies). Labeled proteins were purified from the free dye using Zeba Spin Desalting columns (89882, Thermo Scientific). During all runs, the concentration of labeled molecules was kept constant at 10nM. Recombinant NPTN was titrated over a range of concentrations from 0.122-1000nM. All experiments were done

using the Monolith NT.115 Premium Capillaries (MO-K025) and MST buffer (10mM Na-phosphate buffer, pH7.4, 1mM $MgCl_2$, 3mM KCl, 150mM NaCl and 0.05% Tween-20). MST measurements were done using the Monolith NT.115 instrument with MST power at low setting, exitation power at 100%.

Transfection and plasmids

Np55-EGFP and Np65-EGFP were generously gifted from Dr. Karl-Heinz Smalla (Leibniz Institute for Neurobiology). Lentivirus constructs expressing shRNA were obtained from the Genome Institute of Washington University in St. Louis. The shRNA target sequences are as follows: shGFP, CGACGTAAACGGCCACAAGTT; human shNPTN, CATGGAGTACAGGATCAATAA.

Primary MEFs cultures

Primary MEFs cultures were obtained from WT or *Nptn*-/- mice embryos at E13.5 or 14.5. Their limbs were collected for genotyping before digestion. The embryos were finely minced after dissecting out the head and visceral tissue. Minced tissue was digested in 0.05% Trypsin / EDTA (ThermoFisher Scientific), and isolated MEFs were grown in the medium described above. MEFs were used for experiments within three passages.

Quantitative real-time PCR

Total RNA was isolated from the cells using RNeasy Mini Kit (Qiagen) according to manufacture's instruction. Purified RNA was reverse transcribed to cDNA using ImProm-II

Reverse Transcription System (Promega). Quantitative real-time PCR used was performed on a ViiA 7 Real-Time PCR System (Applied Biosystems) using PowerUp SYBR Green Master Mix (Applied Biosystems). The relative expression for each gene was measured by the standard curve method and normalized against β-actin expression.

Luciferase Assay

pGL4.32[luc2P/NF-kB-RE/Hygro] (Promega) and pRL-TK (Promega) were used for reporter assay. Cells were cotransfected with plasmids/siRNA and reporters as indicated. After 48 hours, the cells were treated with the indicated reagents. Luciferase activity was evaluated by the Dual-Luciferase Reporter Assay System (Promega) according to the manufacturer's protocol. Values obtained from Firefly luciferase signals were normalized to Renilla luciferase activity.

Measurement of apoptosis

Cells were plated to 96 well plates and pre-treated with or without MANF peptide at indicated concentrations for 1 hour. After pre-treatment, the cells were treated with the indicated reagents. Caspase 3/7 activity was then detected using the Caspase-Glo® 3/7 Assay (Promega) using the Infinite M1000 plate reader (Tecan).

Measurement of IL-6

Cells were plated to 24 well plates and transfected with plasmids/siRNA. After 48 hours, IL-6 produced in culture medium was measured with an IL-6 Rat ELISA Kit (ThermoFisher Scientific) according to the manufacturer's protocol.

Statistical analysis

Statistical analysis of the data was performed by one-way analysis of variance (ANOVA) followed by Dunnet's test or Student's t-test using SPSS 22 (IBM).

Cells

HeLa and C6 cells were grown in Dulbecco's Modified Eagle's Medium (DMEM, ThermoFisher Scientific), supplemented with 10% FBS (ThermoFisher Scientific), penicillin (100 U/ml, ThermoFisher Scientific) and streptomycin (100 μg/ml, ThermoFisher Scientific). MEFs were grown in the same medium additionally containing 1% MEM Non-Essential Amino Acids (NEAA ThermoFisher Scientific). INS-1 832/13 cells were grown in Roswell Park Memorial Institute (RPMI) 1640 Medium (ThermoFisher Scientific), supplemented with 10% FBS, 1mM sodium pyruvate (ThermoFisher Scientific), 100 μM β-mercaptoethanol (SIGMA), penicillin, and streptomycin. INS-1E cells were grown in RPMI 1640 Medium, supplemented with 10% FBS, 10mM HEPES, 1mM sodium pyruvate, 1% NEAA, 50 μM β-mercaptoethanol, penicillin, and streptomycin. All kinds of cells were maintained in a humidified atmosphere containing 5% CO2.

Animals

NPTN deficient mice were obtained from INFRAFRONTIER (Strain name: C57BL/6N-Nptn<tm1a(EUCOMM)Hmgu>/H). Animal experiment was performed according to procedures approved by the Institutional Animal Care and Use Committee at the Washington University School of Medicine

KEY RESOURCES TABLE

REAGENT or RESOURCE	SOURCE	IDENTIFIER
**Antibodies**		
polyclonal sheep anti-mouse NPTN	R&D Systems	Cat# AF7818, RRID:AB_2715517
monoclonal mouse anti-human NPTN	Novus Biologicals	Cat#NBP1-47374, RRID:AB_10010327
polyclonal rabbit anti-SLC44A3	GeneTex	Cat# GTX46769, RRID:AB_11172502
polyclonal rabbit anti-MANF	Abnova Corporation	Cat# PAB13301, RRID:AB_10546841
monoclonal rabbit anti-BiP	Cell Signaling	Cat# 3177, RRID:AB_2119845
monoclonal mouse anti-IκB	Cell Signaling	Cat# 4814, RRID:AB_390781
monoclonal rabbit anti-GAPDH	Cell Signaling	Cat# 2118, RRID:AB_561053
monoclonal mouse anti-GFP	MBL	Cat# M048-3, RRID:AB_591823
**Chemicals, Peptides, and Recombinant Proteins**		
Tunicamycin	Sigma	Cat# T7765
Thapsigargin	Sigma	Cat# T9033
Staurosporin	Sigma	Cat# S6942
Recombinant Human MANF	Peprotech	Cat# 450-06
Recombinant Rat IL-1 β	R&D Systems	Cat# 501-RL
Recombinant Rat IFN-γ	R&D Systems	Cat# 585-IF
Streptavidin-HRP	Cell Signaling	Cat# 3999
FITC Streptavidin	BD	Cat# 554060
**Critical Commercial Assays**		
CaptiRec	Dualsystems Biotech AG	Cat# P05201
Caspase-Glo® 3/7 Assay System	Promega	Cat# G8090

IL-6 Rat ELISA Kit	Thermo Fisher Scientific	Cat# BMS625
Dual-Luciferase® Reporter Assay System	Promega	Cat# E1910
RNeasy Mini kit	QIAGEN	Cat# 74104
High-Capacity cDNA Reverse Transcription Kit	Thermo Fisher Scientific	Cat# 4368814
Powerup TM SYBR TM Green	Thermo Fisher Scientific	Cat# 25742
**Oligonucleotides**		
rat *β-actin* RV	Integrated DNA Technologies	GCAAATGCTTCTAGGCGGAC
rat *β-actin* RV	Integrated DNA Technologies	AAGAAAGGGTGTAAAACGCAGC
rat Nptn FW	Integrated DNA Technologies	GCGCCAGAGAAACACAAATTAAG
rat Nptn RV	Integrated DNA Technologies	GCATGCTTTAGACGGTCATTG
rat Il-6 FW	Integrated DNA Technologies	TAGTCCTTCCTACCCCAACTTCC
rat Il-6 RV	Integrated DNA Technologies	TTGGTCCTTAGCCACTCCTTC
rat Cxcl-1 FW	Integrated DNA Technologies	CATTAATATTTAACGATGTGGATGC GTTTCA
rat Cxcl-1 RV	Integrated DNA Technologies	GCCTACCATCTTTAAACTGCACAAT
rat Bip FW	Integrated DNA Technologies	TGGGTACATTTGATCTGACTGGA

rat Bip RV	Integrated DNA Technologies	CTCAAAGGTGACTTCAATCTGGG
rat Chop FW	Integrated DNA Technologies	AGAGTGGTCAGTGCGCAGC
rat Chop RV	Integrated DNA Technologies	CTCATTCTCCTGCTCCTTCTCC
rat Manf FW	Integrated DNA Technologies	TGAGGTATCGAAGCCTCTGG
rat Manf RV	Integrated DNA Technologies	AAGAAAGGGTGTAAAACGCAGC
mouse β-actin FW	Integrated DNA Technologies	GCAAGTGCTTCTAGGCGGAC
mouse β-actin RV	Integrated DNA Technologies	AAGAAAGGGTGTAAAACGCAGC
mouse Il-6 FW	Integrated DNA Technologies	CTCTGCAAGAGACTTCCATCCA
mouse Il-6 RV	Integrated DNA Technologies	AGTCTCCTCTCCGGACTTGT
**Software and Algorithms**		
SPSS 22	IBM	https://www.ibm.com/support/pages/spss-statistics-220-available-download
Flowjo X 10.0.7r2	BD	https://www.flowjo.com/solutions/flowjo
Image Lab	BIO-RAD	https://www.bio-rad.com/en-us/product/image-lab-software
